# The impact of the first United Kingdom COVID-19 lockdown on environmental air pollution, digital display device use and ocular surface disease symptomatology amongst shielding patients

**DOI:** 10.1038/s41598-022-24650-3

**Published:** 2022-12-02

**Authors:** Alberto Recchioni, Maryam Makanvand, Natraj Poonit, Graham R. Wallace, Suzanne Bartington, William Bloss, Saaeha Rauz

**Affiliations:** 1 Academic Unit of Ophthalmology, Institute of Inflammation and Ageing, University of Birmingham, Birmingham, UK; 2 Academic Unit of Ophthalmology, Birmingham and Midland Eye Centre, Birmingham, Institute of Inflammation and Ageing, College of Medical and Dental Sciences, University of Birmingham, Dudley Road, Birmingham, UK; 3 Optometry and Vision Sciences Group, School of Life and Health Sciences, Aston University, Birmingham, UK; 4 School of Geography, Earth and Environmental Sciences, University of Birmingham, Birmingham, UK; 5 Institute of Applied Health Research, University of Birmingham, Birmingham, UK

**Keywords:** Environmental impact, Eye manifestations, Conjunctival diseases, Corneal diseases, Lacrimal apparatus diseases, Vision disorders, Outcomes research, Autoimmunity, Inflammation, Climate-change impacts, Socioeconomic scenarios, Environmental economics

## Abstract

Worldwide lockdown reduced air pollution during the first phase of the COVID-19 pandemic. The relationship between exposure to ambient air pollution, digital display device use and dry eye symptoms amongst patients with severe ocular surface disease (OSD) were considered. Symptoms and air pollutant concentrations for three different time periods (pre, during and post COVID-19 lockdown) were analysed in 35 OSD patients who achieved an immunosuppression risk-stratification score > 3 fulfilling the UK Government criteria for 12-week shielding. OSDI symptoms questionnaire, residential postcode air pollution data obtained from the Defra Automated Urban and Rural monitoring network for concentrations of nitrogen dioxide (NO_2_), nitrogen oxides (NOx), particulate matter (PM) with diameters below 10 µm and 2.5 µm, and English Indices of Deprivation were analysed. Significant reductions in NO_2_ and NOx concentrations were observed between pre- and during-lockdown periods, followed by a reversal in the post-lockdown period. Changes were linked to the Living Environment outdoor decile. A 12% increase (*p* = 0.381) in symptomatology during-lockdown was observed that reversed post-lockdown by 19% (*p* = 0.144). OSDI scores were significantly correlated with hours spent on digital devices (*r*^2^ = 0.243) but not with air pollutant concentrations. Lockdown measures reduced ambient air pollutants whilst OSD symptomatology persisted. Environmental factors such as increased time indoors and use of bluescreen digital devices may have partly played a role.

## Introduction

The Severe Acute Respiratory Syndrome Coronavirus 2 (SARS-CoV-2) global pandemic changed many aspects of our societal behaviour during 2020 and beyond. In the United Kingdom (UK), the government imposed the first national lockdown on 23 March 2020 asking the general public to “stay-at-home” other than for essential journeys. This action resulted in an increase in the time spent indoors^1^ and a reduction of motor vehicle usage by 52–65%^2,3^ between 16 March and 28 April 2020 time periods.

Air pollution is a mixture of gasses and particles present in the air at concentrations known to be damaging to public and environmental health^4^. Two key pollutants within the UK (amongst others) are NO_2_ and particulate matter (PM) where major sources of emissions include road transport, energy generation, industrial combustion, and combustion of wood, coal and solid fuels (DEFRA 2019). Changes in economic activities and travel restrictions during the national lockdown period led to significant reductions in air pollutant emissions, and substantive changes in some ambient air pollutant concentrations (although these were determined both by changes in emissions and meteorology)^5,6^. Levels of nitrogen dioxide (NO_2_) were reduced by up to 42% in urban areas, reflecting local road traffic emission reductions^7^. Similarly, ozone concentrations in urban areas increased reflecting reduced urban decrement^8^. A more complex response was observed in levels of particulate matter (PM_10,_ with an aerodynamic diameter below 10 μm, and PM_2.5_, with a diameter below 2.5 μm), reflecting a wider range of sources and longer atmospheric lifetime. PM_2.5_ concentrations in some locations increased slightly compared to the previous year^9^ corresponding to differences in meteorology and long-range transport^8^. Whilst the “stay-at-home” policy protected the world’s population from exposure to environmental circulation and transmission of SARS-CoV-2, the indoor restrictions led to increased exposure to indoor air pollutants^10^. While ambient (outdoor) pollution levels are key determinants of indoor air quality, the most modern housing is increasingly air-tight to conserve energy reducing not only heat loss, but also ventilation thereby introducing increased exposure to indoor generated pollutants such as NOx and PM from combustion and cooking, and Volatile Organic Compounds (VOC) or Volatile Care Products (VCP) commonly found in toiletries, perfumes and cleaning products^11^. Paradoxically, some pollutants may be present at higher concentrations indoors compared to outdoors, and these in turn have an impact on health^12^. The causal relationship between air pollution and health has long been established, especially, stroke^13^, heart disease^14^, lung cancer^15^ and both chronic and acute respiratory diseases^16,17^. At the eye level, Tear Film Ocular Society Dry Eye Work Shop II in 2017 (TFOS DEWS II) flagged air pollution as an important area for further research^18^, and there is emerging awareness that real-world factors (air conditioning, wind, reading, low humidity, watching television, pollution) are key triggers of exacerbations of disease^19^. Some studies, however, have shown that Dry Eye Disease (DED) symptoms are not associated with air pollutants such as NO_2_^20^, PM_10_^21^, PM_2.5_^20^ and O_3_^22^. Alves, et al.^23^ described the term Environmental Dry Eye Disease (EDED) where dry eye disease is caused by pollutants and/or adverse climatic conditions. Bourcier, et al.^24^ reported data from 3042 patients seen at the emergency department in 1999. High levels of air pollutants were linked with the increased demand for ophthalmological assessment (mainly conjunctivitis and ocular surface disorders) and the authors suggested that further studies with newer air pollution models should be considered to address these connections. More recently, Hao et al.^25^ reported data from 387 DED patients from 5 different provinces across China: increased PM_2.5_ and O_3_ showed increased OSDI scores, reduced tear film stability, upregulated tear inflammatory markers (cytokines) and meibomian gland dysfunction (eyelids secretory glands). In Europe, the cross-sectional association study performed by Vehof et al.^26^ that included 79.866 voluntary participants showed that residential air pollution (NO_2_) could play a role as an independent risk factor. Wolffsohn et al.^27^ reported increased screen exposure can be associated with increased DED symptoms; in fact, increased screen exposure affects the reflex blinking by reducing its frequency and completeness that controls the release of lipid secretion crucial for delaying tear film evaporation. However, the results considering blue light screen and DED are controversial: in-vitro studies showed that blue light affect corneal epithelial cells in culture^28,29^ while Talens-Estarelles et al.^30^ showed that no benefits were observed in subjects before and after 20 min on a laptop computer in terms of DED symptomatology, tear meniscus height, tear film stability and bulbar redness. These findings suggests that further studies are required to clarify this relationship.

During the first UK national lockdown, ocular surface disease patients treated with systemic immunosuppression who achieved a risk stratification score > 3 (Supplementary Table 1) were defined as coronavirus high-risk. These patients were classified as clinically extremely vulnerable (CEV) and fulfilled the government criteria. Clinicians and General Practitioners (GPs) advised people at high-risk to protect themselves by not leaving their homes and minimising all face-to-face contact (*shielding*) for a minimum of 12 weeks^31^. Whilst protected from the outdoor environment, the “stay-at-home” Government policy may have increased the exposure of patients to indoor air pollutants. Our aim was to evaluate whether lockdown-induced changes to air pollution levels influenced dry eye symptomatology amongst these patients by considering three different time periods: “pre”, “during” and “post” lockdown; and whether there was an association with the use of bluescreen digital devices, with the Index of Multiple Deprivation (IMD) and the Living Environment scale and its subdomains that measure ‘outdoor’ and ‘indoor’ living environments as proxy measures of quality of housing and air quality/road traffic, respectively. Following the Homes and Communities Agency (a non-departmental public body that regulates dwellings in England), the quality of housing is based on ten indicators: location, site–visual impact, layout and landscaping, site–open space, site–routes and movement, unit–size, unit–layout, unit–noise, light, services and adaptability, unit–accessibility within the unit, unit–sustainability and external environment.

## Results

Patient demographics are shown in Table 1.

**Table 1:** Demographics of the subjects included in the study.

Patients, n			35
Age, mean ± SD, median, range (years)			69.0 ± 11.0, 70, 42–85
Gender, Female:Male, (%F:%M)			17:18, (49:51)
Ethnicity, n (%)	White—English, Welsh, Scottish, Northern Irish or British		31 (88%)
	White—Irish		1 (3%)
	Asian or Asian British—Indian		1 (3%)
	Black, African, Caribbean or Black British—Caribbean		1 (3%)
	Asian or Asian British—Any other Asian background		1 (3%)
Diagnose, n (%)	Ocular mucous membrane pemphigoid		23 (66%)
	High-risk corneal transplant recipients		3 (9%)
	Peripheral ulcerative keratitis		4 (11%)
	Stevens-Johnson syndrome		2 (5%)
	Granulomatous polyangiitis		1 (3%)
	Sjögren’s syndrome		1 (3%)
	Ocular pemphigus vulgaris		1 (3%)
Index of Deprivation (decile)	Index of Multiple Deprivation, mean ± SD	(Decile 1 to 5 most deprived, decile 6 to 10 least deprived)	5 ± 3
	Living Environment, mean ± SD		5 ± 3
	Indoor, mean ± SD		4 ± 2
	Outdoor, mean ± SD		6 ± 3

The derived concentrations (all in units of μgm^-3^) of NO_2_ and NOx at the study population locations showed a significant reduction of 35% and 44%, respectively (NO_2_ “pre” 17.11 ± 6.87 versus “during” 11.17 ± 4.79 and NOx “pre” 26.06 ± 11.64 versus “during” 14.53 ± 7.18). Together, a significant increase (*p* < 0.001) of particulate matter concentrations was observed between “pre” and “during” lockdown periods (PM_10_ ↑25%, from 13.21 ± 1.28 to 16.54 ± 2.01 μgm^-3^ and PM_2.5_ ↑24%, from 9.06 ± 1.21 to 11.26 ± 1.65 μgm^-3^).

Comparing “pre-lockdown” and “during-lockdown” periods, OSDI ↑12% was noted (36.11 ± 16.09 vs 32.24 ± 29.17, *p* = 0.381); followed by OSDI ↓19% between the “during” and “post” periods (36.11 ± 16.09 vs 29.46 ± 26.29, *p* = 0.144). There were no significant differences in the OSDI sub-domains of vision-related function, ocular symptoms and environmental triggers across all the periods considered.

Multiple correlations were explored between dry eye symptoms and pollutants across all the considered periods, although without significance (Table 2).

**Table 2:** Correlations dry eye symptoms (OSDI questionnaire scores) and air pollutants.

Correlations (n = 35)	NO_2_	NO_x_	PM_2.5_	PM_10_
OSDI PRE *r* [*p-value*]	− 0.023 [0.897]	− 0.022 [0.896]	− 0.07 [0.689]	− 0.068 [0.791]
OSDI DURING *r* [*p-value*]	− 0.124 [0.477]	− 0.127 [0.469]	− 0.151 [0.387]	− 0.169 [0.394]
OSDI POST *r* [*p-value*]	− 0.114 [0.567]	− 0.122 [0.431]	− 0.089 [0.614]	− 0.091 [0.387]

**Figure 1:**
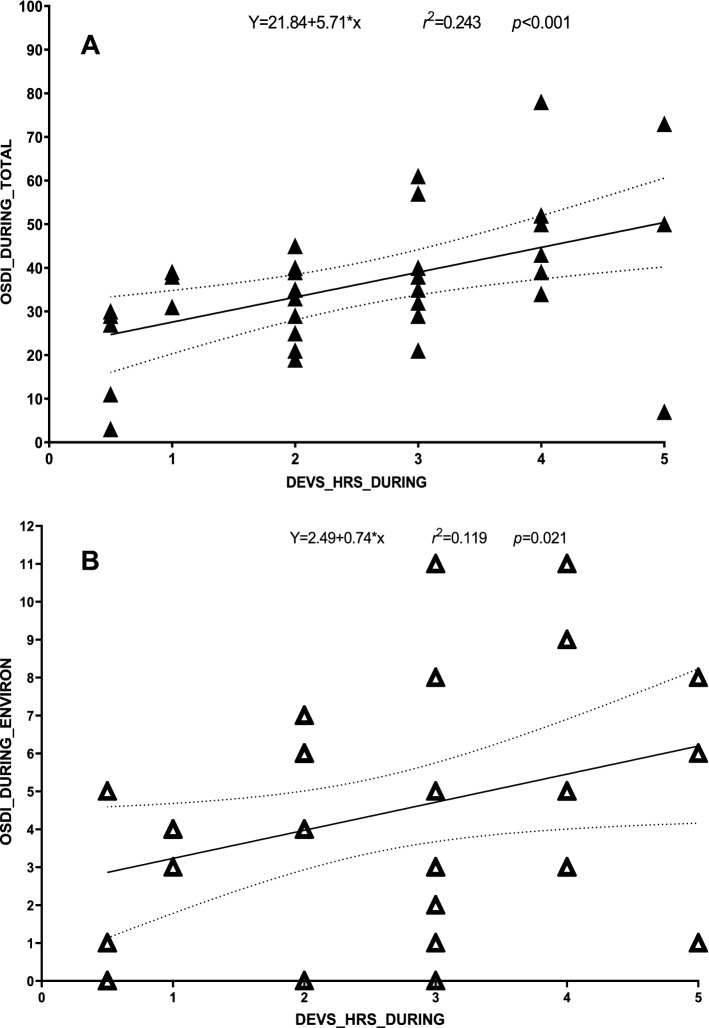
Regression equations: these scatterplots represent the relationships between daily number of hours spent on viewing electronic devices and OSDI questionnaire TOTAL (A) and OSDI questionnaire ENVIRON (B) scores in the “during” period. Dotted lines representing the 95% Confidence Intervals.

Only the correlations observed in the *during* period, between daily number of hours spent on viewing electronic devices (pre/during/post: 2 ± 1/3 ± 1/2 ± 1) and OSDI questionnaire (TOTAL and ENVIRON) scores were significant and therefore represented below: TOTAL (*r*(35) = 0.493, *p* < 0.001), ENVIRON (*r*(35) = 0.345, *p* = 0.021). The assumption for a simple linear regression considered with the residual scatterplot was considered. The relationship between daily number of hours spent on viewing electronic devices and OSDI TOTAL and ENVIRON questionnaire scores were positive and did not reveal any bivariate outliers. The regression equation for predicting the OSDI TOTAL questionnaire scores from daily number of hours spent on viewing electronic devices was *y* = 21.84 + 5.71*x. The *r*^*2*^ for this equation was 0.243; that is 24.3% of the variance in OSDI TOTAL questionnaire scores were predictable from daily number of hours spent on viewing electronic devices (Fig. 1A). The regression equation for predicting the OSDI ENVIRON questionnaire scores from daily number of hours spent on viewing electronic devices was *y* = 2.49 + 0.74*x. The *r*^*2*^ for this equation was 0.119; *i.e*. 11.9% of the variance in OSDI ENVIRON questionnaire scores was predictable from the daily number of hours spent on viewing electronic devices (Fig. 1B).

Most of the participants belonged to the range 1 to 5 decile (most deprived) for the IMD (54%), Living Environment (57%) and Indoor indices (71%); except for the Outdoor index where only 37% belong to the most deprived decile. However, all the air pollutants considered across all the time periods were negatively correlated with the Outdoor index (subscale of the Living Environment index) (Table 3).

**Table 3:** Correlations outdoor index (subscale of the Living Environment index) and air pollutants.

Correlations (n = 35)	PRE Outdoor	DURING Outdoor	POST Outdoor
PM10 r [p-value]	− 0.638 [0.001]	− 0.692 [0.001]	− 0.677 [0.001]
NO2 r [p-value]	− 0.728 [0.001]	− 0.685 [0.001]	− 0.747 [0.001]
NOx r [p-value]	− 0.726 [0.001]	− 0.683 [0.001]	− 0.745 [0.001]
PM2.5 r [p-value]	− 0.747 [0.001]	− 0.713 [0.001]	− 0.752 [0.001]

### Discussion

In 2020, the UK Government imposed the first national lockdown on 23rd March to help to reduce the transmission and health service burden of SARS-CoV-2.

This led many hospitals and clinicians to inform high-risk patients to confine at home posing a new challenge for ophthalmic practitioners specialising in ocular surface and DED patients. In fact, the number of face-to-face consultations reduced by up to 65%^32^ to avoid spreading the SARS-CoV-2 disease and most follow-up visits were converted to virtual or telephone consultations. One of the most common diagnostic tools in dry eye disease, in particular during the pandemic, has been the Ocular Surface Disease Index (OSDI) questionnaire which is an established instrument to track the severity of the disease^33^. Previously, Amparo and Dana^34^ demonstrated that dry eye symptoms can be virtually monitored using the OSDI questionnaire without asking the patients to physically attend the clinic. Inomata et al.^35^ considered the OSDI questionnaire to validate the use of electronic crowdsourced data in distinguishing undiagnosed vs diagnosed dry eye patients in a cross-sectional study of more than 4454 patients. The authors were able to demonstrate that telemedicine and its related data collection could play a role in the DED follow-up. Our OSDI score results showed a significant increase in symptoms when comparing “pre” versus “during” lockdown periods while no significant change was found comparing other periods. This change may be attributed to different environmental factors experienced by the patients during the lockdown confinement. These may include the psychological impact of the lockdown which reduced the number of social interactions and increased patients’ seclusion. In fact, DED has been shown to be associated with psychological disorders such as anxiety, depression and post-traumatic stress disorder^36^. Another important factor that might have increased symptoms in the shielding patients included in the study is time spent with electronic devices. Studies have shown how the eye can be affected by the use of digital screens leading to side effects such as eye strain, ocular dryness, itchy eyes, blurred vision and irritation^37,38^. Despite the small sample size, our study demonstrated that the daily number of hours spent on viewing electronic devices (“pre” 1.80 ± 0.80 h versus “during” 2.50 ± 1.38 h) may have accounted for up to 24.3% of the variance in OSDI questionnaire results comparing “pre” versus “during” (r(33) = 0.493, *p* < 0.001), and the linear regression showed that an increased time spent with electronic devices is statistically correlated with dry eye symptoms increase. Additionally, the environmental domain which includes OSDI questions related to low humidity and air-conditioning (ENVIRON) was influenced up to 11.9% by the daily number of hours spent on viewing electronic devices confirming other studies where a relationship between these factors was observed^37,39^.

The relationship between DED and air pollution has previously been explored: Torricelli et al.^40^ reported that a sample of 71 taxi drivers and traffic controllers exposed to high levels NO_2_ and PM_2.5_ showed a reduction of tear film stability (TBUT) and influences over the tear film osmolarity. Similar findings were presented by Gupta et al.^41^ for an urban population where 24% of the subjects presented a reduced TBUT versus only 5.2% living outside the urban area. This work reports similar findings to Torricelli’s^40^and Berg’s studies^42^ where no correlations were found between OSDI questionnaire scores and air pollutant levels. Apparently, it seems evident that the increased symptoms may be linked with increased levels of atmospheric pollution^42^ but not the converse: in our case a significant increase of 12% OSDI questionnaire scores when comparing “pre” versus “during” lockdown periods where NO_2_ and NO_X_ reduced up to 44%.Torricelli’s study^40^ noted that the levels of pollutants reported (PM_2.5_ and NO_2_) were at least 3 and 10 times higher than found here, and that even at such levels, were not correlated with patient symptoms. We can therefore speculate that other environmental factors might have played a role in the symptoms increase. For example, we could speculate that the forced indoor confinement has exposed patients’ ocular surface to a diminished level of humidity, that even in presence of a reduction of air pollutants such as NO_2,_ has manifested an increase in their symptoms. Additionally, we might hypothesize that the potential rise of stress and anxiety due to the confinement could have led to a dysregulated diet in favour of unhealthy foods such as fried meals, refined sugars and artificial sweeteners instead of vegetables rich in vitamins. Unfortunately, we were not able to measure indoor humidity levels in patients’ homes or any diet variation during the home confinement which might have skewed our results.

Living in poorer areas has a higher risk of morbidity and mortality^43^. It has been shown that living in the most deprived area may affect eye health and the prevalence of certain eye diseases such as diabetic retinopathy^44^, glaucoma^45^ and age-related macular degeneration^46^. By comparison, very little is known about the relation between DED and indices of deprivation. For a matter of consistency, along with this research, we have considered the latest data available in 2019 considering the overall IMD decile, Living Environment decile and its two subdomains: indoor and outdoor deciles. We reported several significant correlations (*r* range between −0.638 and −0.752) between the air pollutant readings and the outdoor decile only. As expected, these results demonstrated that the most deprived areas (deciles 1 to 5) were the ones with the worst scores (air quality and road traffic accidents), even during lockdown periods, although our results showed no correlations between dry eye symptomatology and these indices. These contrasting results are difficult to explain, but as mentioned earlier, it may be due to a lack of correspondence between the OSDI questionnaire and air pollution levels^40,42^ or due to the lack of correlation between indoor and outdoor pollution^47^.

The main limitation of our study is the small sample size and that it necessarily lacks of objective dry eye tests because the study population fulfilled the government criteria for shielding for a minimum of 12 weeks. Documented risk factors of dry eye severity such as smoking, unbalanced diet, diabetes were not investigated in this study. Additionally, we were not able to verify that the time spent with electronic devices was observed with an appropriate and updated refraction (e.g. spectacles), under controlled visual ergonomics (e.g. distance, illumination and posture) or if the devices considered were equipped with or without blue light filter technology. A further limitation is that indoor air pollution was not directly measured. Inclusion of such factors would develop this research further and give a better understanding of patient exposure, especially since individuals spend more time indoors^12^. In some cases, indoor exposure to pollutants can be higher to that outdoors and so could give an alternative explanation as to an increase of DED when indoors. Previous studies have shown an increase in DE symptoms associated to increased exposure of indoor O_3_^48^ and VOCs^49^. It is also recognised that social and economic changes occurred in response to the emerging pandemic prior to introduction of formal public health measures on 23rd March 2020, and therefore our pre-lockdown phase may not completely reflect typical baseline environmental conditions.

This is the first specific study to our knowledge to evaluate whether the first 2020 UK national lockdown measures changed the ambient air pollution level/DED symptomatology relationship amongst shielding patients. We explore how other environmental factors, such as indices of deprivation and time spent with electronic devices, might had a role on patients’ DED symptoms. The limitations of the present study indicate that the results should be interpreted with caution and further studies of DED, air pollution exposure, digital display use and indices of deprivation are recommended.

## Methods

The research was carried out in accordance with the tenets of the Declaration of Helsinki and was approved by the Regional Ethical Committee Sandwell and West Birmingham NHS Trust Department of Clinical Effectiveness (protocol number and registration #1611). Informed consent was obtained from each patient.

### Study population

Eighty patients with severe ocular surface disease maintained on systemic immunosuppression achieved a risk stratification score of > 3 defined as coronavirus ‘high-risk’, fulfilled the UK government criteria for shielding for a minimum of 12 weeks, were identified (Supplementary Table 1). Only thirty-five patients decided to take part in the study (Table 1) giving a response rate of approx. 44%. Immunosuppression regimens consisted of mycophenolate mofetil, azathioprine, tacrolimus, methotrexate and cyclophosphamide.

### Time period

Three time periods were defined as: “Pre” December 2019–March 2020 (01/12/2019 to 23/03/2020), “During” April 2020–May 2020 (01/04/2020 to 10/05/2020), and “Post-lockdown” June–July 2020 (15/06/2020 to 18/07/2020).

### Dry eye symptoms

“Pre” lockdown symptoms were curated from hospital electronic databases that used the OSDI symptoms questionnaire (Allergan plc, Irvine, CA), whilst “during” and “post” lockdown data were obtained via postal hardcopy of the OSDI. Information on the daily number of hours spent on viewing electronic devices by the patients were also collected across all the periods. All the OSDI domains such as vision-related function (VISFUNCT), ocular symptoms (OCSYMP) and environmental (ENVIRON) triggers across all the periods were taken into account. A bivariate regression was conducted to determine how time spent on digital bluescreen devices (such as smartphones, tablets and computers) could predict the level of symptomatology based on the OSDI scores.

### Index of multiple deprivation, living environment and indoor and outdoor subscales

The English Indices of Deprivation 2019 (IoD2019) is the official measure of relative deprivation for geographical areas in England and it is comprised of seven distinct domains of deprivation which, when combined and appropriately weighted, form the Index of Multiple Deprivation (IMD2019). These 7 different domains are: Income Deprivation, Employment Deprivation, Education, Skills and Training Deprivation, Health Deprivation and Disability, Crime, Barriers to Housing and Services and Living Environment Deprivation.

Full residential postal codes were used to extract indices of deprivation from IoD 2019 considering the latest available database at the time of conducting the study: https://www.gov.uk/government/statistics/english-indices-of-deprivation-2019 and https://imd-by-postcode.opendatacommunities.org/imd/2019. Specific data extracts were obtained from the IMD and the Living Environment Deprivation decile for ‘indoor’ and ‘outdoor’ sub-domains. The “indoor” index measures the quality of housing that considers ten different indicators such as location, site–visual impact, layout and landscaping, site–open space, site–routes and movement, unit–size, unit–layout, unit–noise, light, services and adaptability, unit–accessibility within the unit, unit–sustainability and external environment. The “outdoor” index contains measures of air quality and road traffic accidents.

### Air quality

Air quality data associated with patient residential postcodes were derived from Department of Food and Rural Affairs (DEFRA) datasets. Defra provides annual mean estimated background concentration maps which are primarily used to support the Local Air Quality Management (LAQM) Review and Assessment process for Local Authorities. These estimated concentrations are provided on a 1 km grid resolution for the whole of the UK. DEFRA also operates a network of air quality monitoring stations, notably the Automatic and Urban Rural Network (AURN), providing high time resolution (hourly) data at specific locations (there are 13 background AURN stations used within the study area considered here). Ambient air quality exposure change during lockdown for each patient location was estimated by combining the geographical variation from the Defra background maps with the temporal variability as measured by the AURN stations.

Each patient’s residential postcode involved in the study were allocated Defra annual concentrations for PM_10,_ NO_2_, NOx and PM_2.5_, depending on which 1 km grid square the postcode was located in, for the years 2019 and 2020. To adjust the annual mean concentrations to the relevant time periods over which exposure was considered (as air pollutant concentrations vary systematically with season), a time-scale factor was applied, based on the seasonal cycle observed at the nearest AURN station. Patient postcodes were also matched to the nearest background AURN monitor, for each pollutant (PM_10,_ NO_2_, NOx and PM_2.5_). It should be noted that AURN monitored data was filtered to only include background monitors (i.e. to remove the impact of “roadside” measurements which would not reflect residential exposure). This was to eliminate localised concentrations which might affect specific monitors but which would not reflect the patients’ postcodes. A population background pollution level was therefore considered to be the best approach. Some AURN monitors only measure one pollutant. To avoid missing data of the other pollutants, postcodes were matched to next nearest AURN monitor with nearest available data in this case.

Time-scale factors were created for every background AURN site that was included in this study. This was achieved by first obtaining hourly concentrations for NO_2_, NOx, PM_10_ and PM_2.5_ for the background AURN and calculating an annual average and lockdown period mean concentration for each pollutant. Pre-lockdown (December 2019–March 2020), during-lockdown (April–May 2020) and post-lockdown (June–July 2020).

### Statistical analysis

All statistical analyses were performed using SPSS 23.0 (SPSS Inc, Chicago, IL). Data normality was tested using the Shapiro–Wilk test. Group comparisons for the normally distributed data were performed with the Student t test considering the different time points (“pre”, “during” and “post”), whereas the non-normally distributed variables were examined with the Wilcoxon signed rank test with 2 related samples. The bivariate correlation analysis for non-normally distributed data were analysed using the Spearman test. A guide to interpreting the correlation strength was derived from the recommendations of Navarro^50^. A *p* value of 0.05 was taken to be statistically significant. The sample size was calculated using MedCalc version 10.0 (MedCalc, Ostend, Mariakerke, Belgium). The minimum sample size requirement for a t-test with an alpha level of 0.05, and a power of 0.8, was calculated to be a minimum of 21 patients. Finally, a bivariate regression was performed across all metrics included examining any influences between an explanatory variable and an outcome variable.

## Supplementary Information

Supplementary Information.

## Data Availability

The datasets generated during and/or analysed during the current study are available from the corresponding author upon reasonable request.
