# Supplementary material for: The impact of the first United Kingdom COVID-19 lockdown on environmental air pollution, digital display device use and ocular surface disease symptomatology amongst shielding patients
Source: Sci Rep. 2022 Dec 2;12:20820. doi: 10.1038/s41598-022-24650-3 (PMC9716517; doi:10.1038/s41598-022-24650-3)
Supplement: Supplementary file 1 — Supplementary Information. [file 41598_2022_24650_MOESM1_ESM.pdf]

# Inflammatory Eye Disease Service COVID-19 Response: Identifying high-risk patients for shielding and self-isolation

| Risk Stratification Guide | Definite High Risk: To be advised to shield for a minimum of 12 weeks | Moderate Risk: Social Distancing | Low Risk: Social Distancing |
|---|---|---|---|
| Immunosuppressive medication | • Corticosteroid dose of ≥20mg (0.5mg/kg) prednisolone (or equivalent) per day for more than four weeks<br>• Corticosteroid intravenously within the previous 4 weeks<br>• Cyclophosphamide at any dose orally or within last six months if given intravenously<br>• Corticosteroid dose of ≥5mg prednisolone (or equivalent) per day for more than four weeks plus at least one other immunosuppressive medication*, biologic/ monoclonal** or small molecule immunosuppressant (eg JAK inhibitors)***<br>• Any **TWO** agents amongst immunosuppressive medications, biologics/monoclonals** or small molecule immunosuppressants with any co-morbidity**** | • Well-controlled patients with minimal disease activity and no co-morbidities on **SINGLE** agent broad spectrum immunosuppressive medication, biologic/monoclonal** or small molecule immunosuppressant<br>• Well-controlled patients with minimal disease activity and no co-morbidities on **SINGLE** agent broad spectrum immunosuppressive medication plus Sulphasalazine or Dapsone and/ or hydroxychloroquine<br>• Well-controlled patients with minimal disease activity and no co-morbidities on a **SINGLE** agent broad spectrum immunosuppressive medication* at standard dose (eg Methotrexate up to 25mg per week) ***plus*** single biologic** (eg anti-TNF) or small molecule immunosuppressant*** (eg JAKi) | • Single agent 5-ASA medications (eg mesalazine)<br>• Singleagent6-mercaptopurine<br>• Hydroxychloroquine<br>• Doxycycline<br>• Sulphasalazine<br>• Topical immunosuppressant such as ciclosporin drops/ointment, tacrolimus ointment, or topical steroids within 4 weeks<br>• Intravitreal steroids and immunosuppressants within 6 months<br>• Intramuscular steroids within the previous 4 weeks<br>• Topical serum eye drops |

* **Immunosuppressive medications include**: Mycophenolate (mycophenolate mofetil or mycophenolic acid), Azathioprine, Methotrexate, Ciclosporin, Tacrolimus, Cyclophosphamide, Sirolimus, Dapsone. It does **NOT** include Doxycycline, Hydroxychloroquine or Sulphasalazine either alone or in combination

** **Biologic/monoclonal includes**: Rituximab or intravenous immunoglobulin within last 12 months; all anti-TNF drugs (etanercept, adalimumab, infliximab, golimumab, certolizumab and biosimilar variants of all of these), Tocilizumab, Abatacept, Belimumab, Anakinra, Secukinumab, Ixekizumab, Ustekinumab, Sarilumab, Canakinumab

*** **Small molecules include**: all JAK inhibitors – Baracitinib, Tofacitinib etc

**** **Co-morbidity includes**: Age >70, Diabetes Mellitus, any pre-existing lung disease, renal impairment, any history of Ischaemic Heart Disease or hypertension

**NB** This advice applies to both adults, children and young people with rheumatic disease. We do **NOT** advise that patients increase steroid dose if they become unwell



Supplementary Table 1 Inflammatory Eye Disease Service COVID-19 Response: Identifying high-risk patients for shielding and self-isolation (Page **1** of **2)**

UNIVERSITY OF BIRMINGHAM

BMEC Birmingham & Midland Eye Centre Est 1823

Sandwell and West Birmingham Hospitals NHS Trust

# Risk Stratification of Patients with Inflammatory Eye Disease

To be used in conjunction with BMEC-IED_COVID-19_Patient Advice 31 March 2020

**Summary**

- **Score of 3 or more: Patients to shield**
  **Score of <3:**
  - **To exercise once a day either alone, or with members of your household**
  - **Shopping for basic necessities, although this should be done as little as possible**
  - **Medical need or to provide care for a vulnerable person**
  - **Travel to or from work but only where this is absolutely necessary**
  - **Patients to wear eye protection where appropriate**

| Risk Factor | Score |
|---|---|
| • 20mg or more (0.5mg/kg) Prednisolone per day for more than four weeks | **3** |
| • 5mg or more Prednisolone but less than 20mg per day for more than four weeks | **2** |
| • Any Cyclophosphamide within last six months | **3** |
| • 1 drug of immunosuppressive medication* or biologic** | **1** |
| • 2 or more drugs of: immunosuppressive medication* or biologic** | **2** |
| • Any one or more of these: age >70 years, Diabetes Mellitus, lung disease, kidney disease, heart disease or high blood pressure | **1** |
| • Local immunosuppressants such as topical ciclosporin drops/ointment, tacrolimus ointment ortopical steroids; intravitreal injections (steroids, rituximab), orbital floor or periocular steroid injections | **0.5** |
| • Topical serum eye drops | **0** |
| • Hydroxychloroquine, Sulfalsalazine, Dapsone, Doxycycline/Erythromycin alone or in combination | **0** |

* **Immunosuppressive medications include**: Mycophenolate (mycophenolate mofetil or mycophenolic acid), Azathioprine, Methotrexate, Ciclosporin, Tacrolimus, Cyclophosphamide, Sirolimus, Dapsone.

** **Biologic/monoclonal includes**: Rituximab or intravenous immunoglobulin within last 12 months; all anti-TNF drugs (etanercept, adalimumab, infliximab, golimumab, certolizumab and biosimilar variants).

**NB** **Immunosuppressive agents do NOT include:** Doxycycline, Hydroxychloroquine or Sulphasalazine either alone or in combination; or topical immunosuppressants such as ciclosporin drops/ointment, tacrolimus ointment, or topical steroids

**BMEC-IED_COVID-19_Patient Advice** – *adapted from the British Society of Rheumatology 'Advice for immunosuppressed patients' Version 0.08 date 31 March 2020*

Supplementary Table 1 Inflammatory Eye Disease Service COVID-19 Response: Identifying high-risk patients for shielding and self-isolation (Page **2** of **2)**
